# Thanks

**Published:** 1887-10

**Authors:** 


THANKS.—We want to return sincere thanks to our kind friends, Dr. J. H. Bowers, of Columbus, for several new subscribers and for five members of the P. M. B. A.—Drs. E. E. Smiley, Terrell, and E. M. Fowler, of Forney, for a club of subscribers, and the cash—and to numerous other friends for kind and cheering words accompanying their remittances, which have been coming in very freely. Such evidences of appreciation and sympathy are most gratefully acknowledged and esteemed.

